# Effects of tourniquet use on clinical outcomes and cement penetration in TKA when tranexamic acid administrated: a randomized controlled trial

**DOI:** 10.1186/s12891-021-03968-5

**Published:** 2021-01-31

**Authors:** Zeng Yi, Li Yan, Si Haibo, Wu Yuangang, Li Mingyang, Liu Yuan, Shen Bin

**Affiliations:** 1 Department of Orthopaedic Surgery, Orthopaedic Research Institue, and National Clinical Research Center for Geriatrics, West China Hospital, Sichuan University, Sichuan Province, Chengdu, 610041 China; 2 Department of Orthopaedic Surgery, West China Hospital, Sichuan University, 37# Guoxue Road, Chengdu, 610041 People’s Republic of China; 3 Department of Radiology, West China Hospital, West China Medical School, Sichuan University, Sichuan Province Chengdu, 610041 China

**Keywords:** Total knee arthroplasty, Tourniquet, Tranexamic acid, Total blood loss, Bone cement penetration

## Abstract

**Background:**

The role of a tourniquet is still controversial for patients undergoing total knee arthroplasty (TKA). Our current study was performed to determine whether the nonuse of the tourniquet combine with tranexamic acid (TXA) application in TKA patients with end-stage osteoarthritis would accelerate the perioperative recovery rate and provide enough cement mantle thickness for implant fixation.

**Methods:**

In this prospective, randomized controlled trial, 150 end-stage knee osteoarthritis patients receiving TKA were divided into three groups: **group A (tourniquet group), group B (non-tourniquet group), and group C (tourniquet in cementation group)**. All enrolled patients received 3 g of intravenous TXA and 1 g topical TXA. The primary outcomes included blood loss variables and transfusion values. The secondary outcomes included VAS pain score, inflammatory factors level, range of motion, HSS score, postoperative hospital stay, and complication. Furthermore, by using a digital linear tomosynthesis technique, tibial baseplate bone cement mantle thickness was measured in four zones based on the knee society scoring system.

**Results:**

No significant difference was found among the three groups with regards to total blood loss, transfusion, and complication. However, patients in **group B** showed lower inflammatory factors levels, shorter length of hospital stay, better range of motion, and lower postoperative pain. No significant difference was found among the three groups in four zones in terms of bone cement mantle thickness.

**Conclusions:**

For end-stage knee osteoarthritis patients, the absence of tourniquet did not appear to affect blood loss and cement penetration in TKA patients. Furthermore, less inflammation reaction and better knee function can be achieved without a tourniquet. We recommend no longer use a tourniquet in primary TKA for patients with end-stage osteoarthritis when TXA is administrated.

**Trial registration:**

ChiCTR-INR-16009026.

**Level of evidence:**

Therapeutic Level I.

The tourniquet, which is widely used in total knee arthroplasty (TKA), has the proposed benefits of reducing operation time and improved visualization due to reduced bleeding. Several previous studies have proved that a tourniquet application could reduce total blood loss and create a clean blood-poor surface surgical time, which can get a long-term survival rate of cemented TKA components [1–3].

However, the role of a tourniquet is always controversial. Some potential complications have been reported in the literature, including the delayed recovery of quadriceps strength, subcutaneous fat necrosis, increased risk of infection, nerve palsy, and deep venous thrombosis, especially in obese patients [4, 5]. Nowadays, no consensuses have been reached with regard to defining an optimal tourniquet application strategy. The application of tranexamic acid (TXA) in TKA has dramatically improved peri-operative blood management. Numerous studies have proved that TXA significantly decreases postsurgical blood loss and transfusion requirements without increasing the risk of venous thrombosis events (VTEs) [4, 6, 7]. However, seldom evaluated the effect of TXA when patients undergoing TKA without a tourniquet [8].

The purpose of our current randomized controlled study (RCT) was to determine whether intravenous and topical application of TXA in TKA patients without a tourniquet would affect: (1) intraoperative blood loss and total blood loss; (2) postoperative pain, range of knee motion and HSS scores; (3) operation time, length of hospital stay after operation and complications; (4) four zones of tibial side cement mantle thickness based on the knee society scoring system. We hypothesized that TXA combined application without a tourniquet is the best choice for patients undergoing primary TKA, which could accelerate the healing process and provide enough cement mantle thickness for implant fixation, with little or no noticeable side effect.

## Materials and methods

### Study design and participants

This study adheres to CONSORT guideline and was registered in the Chinese Clinical Trial Registry (date of registration: 14/8/2016, registration number: ChiCTR-INR-16009026.), a first-level registration institution of the WHO International Clinical Trial Registry Platform. Approval was obtained from the Clinical Trials and Biomedical Ethics Committee of West China Hospital (No. 201302007) and all methods were carried out in accordance with relevant guidelines and regulations (Declaration of Helsinki). Written informed consent was obtained from all the participants. All the methods were conducted according to the CONSORT 2010 statement.

From May 2017 to June 2018, patients undergoing primary TKA were eligible for this trial. All the patients enrolled were diagnosed with end-stage osteoarthritis (OA). Exclusion criteria included: a history of hypercoagulation, hemophilia, deep vein thrombosis (DVT), pulmonary embolism (PE); previous surgery to the knee; bleeding disorders, platelet of bone marrow disorders; patients were diagnosed with other diseases, such as rheumatoid arthritis; patients with diabetes, peripheral neurovascular disease, malignant tumor; preoperative Hb < 100 g/L. The duration of postoperative follow-up in this study was 3 months.

### Randomization and trial intervention

Recruited patients were randomly divided into three groups using sealed envelopes in a 1:1:1 ratio opened before surgery. All the surgeries were performed by two senior surgeons in the standard way, using a midline skin incision and a standard medial parapatellar approach. All the included patients received TXA using the same strategy: intravenous TXA was applied 5 min before incision (1 g) and 3 (1 g) and 6 (1 g) hours later after the procedure (3 g TXA intravenously in total). One gram of topical TXA in 100 ml of normal saline solution was irrigated in the wound after implantation of the components. During the procedure, in the **group A (tourniquet group)**, patients receiving a full-time tourniquet during the whole procedure. In **the group B (non-tourniquet group)**, TKA was performed without a tourniquet during the whole procedure. **In the group C (tourniquet in cementation group)**, tourniquet was inflated before prosthesis placement and deflated after cement hardened. The tourniquet (VBM, Germany) was applied in 100 mmHg above systolic blood pressure. Controlled hypotension technique was applied in all operations, in which the systolic pressure was controlled in 90–100 mmHg and the diastolic pressure was in 50-60 mmHg.

All the patients received the same prosthesis, a posterior-stabilized fixed bearing P.F.C TKA (DePuy, Warsaw, IN, USA). A total amount of 40 g of bone cement (Smartest GMV Endurance, DePuy, Blackpool, England) was used for all patients.

### Postoperative management

Intra-articular drainage was applied in every patient and removed in the next morning (within 24 h after the operation). 0.3 ml (3000 IU) low molecular weight heparin (LMWH) was started 8 h postoperatively and repeated at 24 h intervals in the subsequent days while in hospital. After discharged, 10 mg active direct factor Xa inhibitor (Rivaroxaban, Xarelto, Bayer Healthcare) was administrated orally once a day and lasted for 2 weeks after operation. Every patient received the same standardized postoperative pain control strategy [9]. Hemoglobin and hematocrit levels were determined on the first and third days after surgery. Active isometric quadriceps and initiative straight-leg raising motion was started just after surgery. Full weight-bearing was permitted since 24 h postoperatively.

### Clinical evaluation

Clinical evaluation included blood loss evaluation and knee rehabilitation measurement. Perioperative blood loss, drainage volume, and total blood loss (calculated using the modified Gross formula [10]) were determined. The blood transfusion rate was also documented. The use of blood transfusions was standardized, which the hemoglobin concentration was < 70 g/L or a patient developed any anemia-related organ dysfunction. Knee pain score, using the visual analogue scale (VAS) method, was documented on the first and third day after surgery. In order to determine whether tourniquet application increase inflammatory reaction and reduce the rehabilitation process after surgery, inflammatory and muscle injury factors (CRP, ESR, IL-6, and CK), range of motion, HSS score were also determined. Furthermore, the length of the postoperative hospital stay and postoperative complications were determined.

### Radiological evaluation

**Fig. 1:**
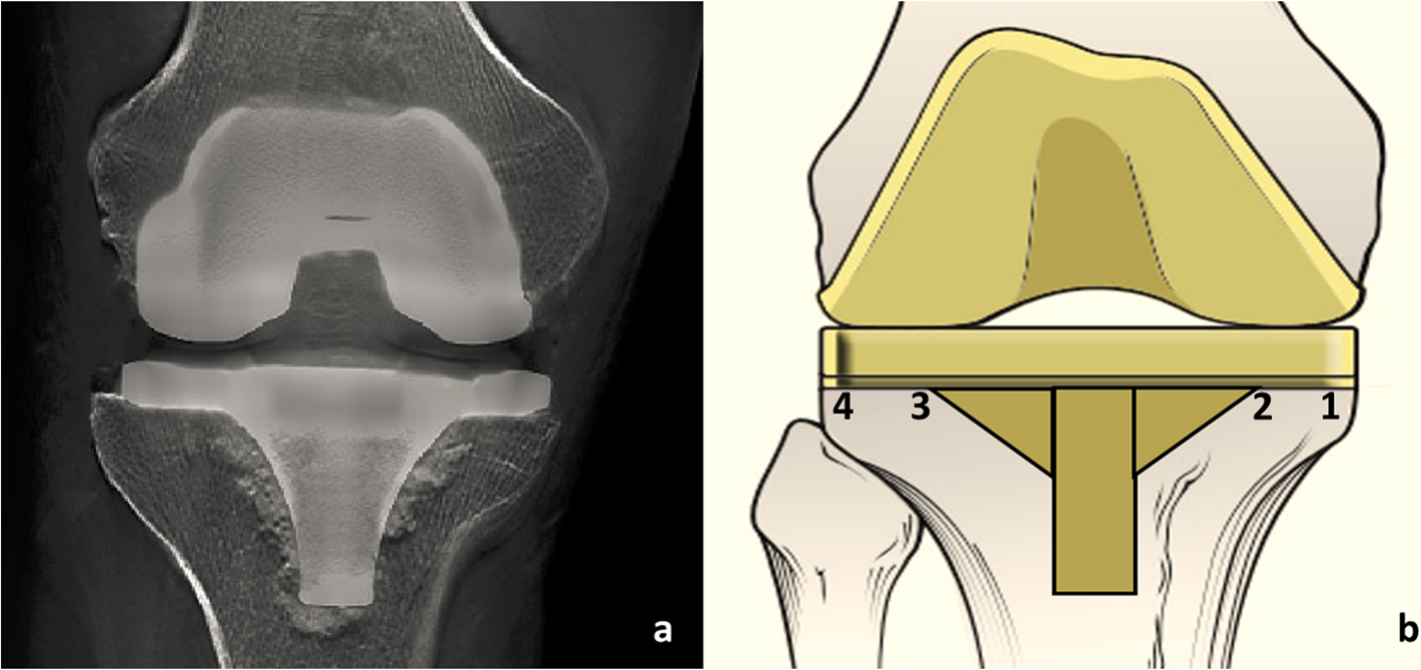
All patients received digital linear tomosynthesis (DTS) examiniation to measure bone cement mantle thickness. The measurement was performed based on the Knee Society scoring system. **a**. The radiographic film of digital linear tomosynthesis (DTS) examination, which showed better contrast than conventional DR film. **b** mantle thickness was measured in tibial baseplate based on the four zones

On the first postoperative day, a standard digital anteroposterior and lateral radiograph of the operated knee was taken, which was used to determine the component position. Furthermore, in order to get accurate data of the cement mantle, all patients received digital linear tomosynthesis (DTS) examination in a standard way [11, 12]. DTS showed greater contrast than conventional DR (Fig. 1a). It gives good results independent of the type of metal and shows good results for the removal of noise artifacts. The effectiveness of this method in enhancing the visibility of a cement mantle was quantified in terms of the signal-to-noise ratio (SNR) and removal of ghosting artifacts in a prosthesis patient. After examination, two radiologists, who blinded whether a tourniquet was used, measured the thickness of the cement mantle independently within the local picture archiving and communication system (PACS). Measurements were performed in four zones based on the Knee Society scoring system and previous surgeons’ experience (Fig. 1b) [7, 13]. Cement mantle thickness was measured only in the tibial baseplate and the results were recorded in centimeters with two decimal [7]. The results of mantel thickness in four zones and total cumulated thickness were compared among groups, respectively.

### Statistical analysis

The statistical analysis was the same as our previous RCT study [14]. The distribution of potential confounders among the study groups and the outcomes were assessed with summary statistics, including the means and standard deviations for quantitative data and the frequencies and percentages for qualitative data. The continuous variables were compared using a one-way analysis of variance between the two groups and the categorical variables were compared using the Pearson chi-square test. The level of statistical significance was set at *P* < 0.05. The statistical analysis was performed using SPSS software (version 13.0; SPSS Inc., Chicago, IL, USA).

## Results

### Patients

**Fig. 2:**
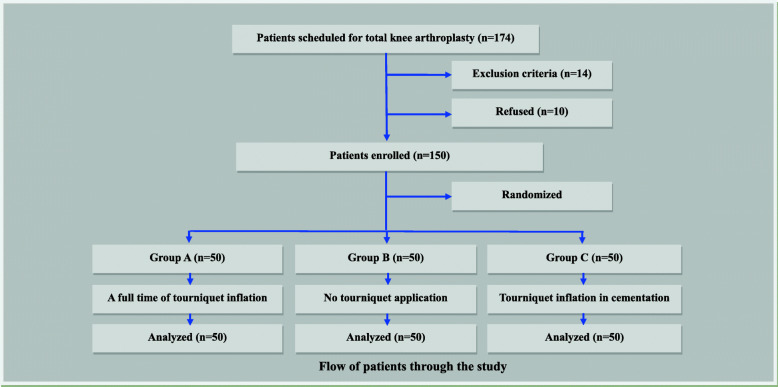
Flow of patients through the study

One hundred seventy four patients were diagnosed with end-stage osteoarthritis and scheduled to have TKA in our hospital. Twenty four patients were not approached for multiple reasons: 14 were ineligible according to our criteria, and 10 declined to participate in the study (Fig. 2). The remaining 150 eligible participants formed the study cohort, with 50 randomized to each group. The three groups were similar at baseline with no significant difference among the groups (Table 1).

**Table 1:** Baseline characteristics of the study population

Variable	Group A (Tourniquet group) (***N*** = 50)	Group B (Non-tourniquet group) (*N* = 50)	Group C (Tourniquet in cementation group) (*N* = 50)	*p* value*
Demographic characteristics				
Age (yr)	68.44 ± 6.80	68.00 ± 7.11	68.66 ± 7.27	0.89
Male sex (%)	7 (14%)	8 (16%)	7 (14%)	–
Weight (kg)	64.39 ± 8.16	62.66 ± 10.44	63.64 ± 11.27	0.67
Height (cm)	153.88 ± 21.49	157.06 ± 6.74	156.52 ± 7.08	0.46
BMI (kg/m^2^)	26.13 ± 2.63	25.34 ± 3.61	25.88 ± 3.51	0.42
ASA status I-II/≥III (No. of patients)	35/15	38/12	36/14	–
Diagnosis				
Primary OA	50	50	50	–
Preoperative laboratory values				
Preo. Hemoglobin (g/dl)	134.84 ± 12.25	132.18 ± 15.10	129.50 ± 14.98	0.17
Preo. Hematocrit (L/L)	0.41 ± 0.04	0.41 ± 0.04	0.40 ± 0.04	0.82
Preo. CRP (10^9^/L)	3.94 ± 3.13	3.29 ± 2.00	3.79 ± 2.30	0.41
Preo. ESR (mm/L)	21.88 ± 12.59	24.08 ± 13.21	28.32 ± 16.87	0.08
Preo. IL-6 (pg/ml)	4.28 ± 2.04	3.40 ± 2.19	3.76 ± 2.27	0.13
Preo. CK (IU/L)	81.52 ± 29.89	85.02 ± 39.71	74.06 ± 31.36	0.26
Preoperative knee function				
VAS score (points)	7.28 ± 0.97	7.32 ± 0.95	7.16 ± 0.87	0.67
**Range of motion**	**94 ± 16**	**97 ± 20**	**95 ± 14**	**0.67**
HHS score (points)	56.64 ± 4.24	55.10 ± 4.62	56.74 ± 4.04	0.11

Abbreviations: OA, osteoarthritis; CRP, C-reaction protein; ESR, erythrocyte sedimentation rate; IL-6, interleukin-6; CK, creatine kinase; HSS score, hospital of special surgery score

*The *p* value represents the result of one-way analysis of variance for independent means for continuous variables or the chi-square test for independent proportions that included the three groups

### Surgical results and complications

Table 2 reveals the results of perioperative assessment results. The mean surgical time in the group A, group B, and group C were 76.70 min, 79.30 min, and 80.70 min, respectively, with no significant intergroup differences. The mean tourniquet time in the group A was 61.00 min, which was significantly longer than those in group C (10.20 min).

**Table 2:** perioperative characteristics of patients utilizing tourniquet or not

Variable	Group A (Tourniquet group) (*N* = 50)	Group B (Non-tourniquet group) (*N* = 50)	Group C (Tourniquet in cementation) group (*N* = 50)	*p* value*
Surgical information				
Surgical time	76.60 ± 8.72	79.30 ± 8.21	80.70 ± 10.15	0.07
Tourniquet time	61.00 ± 6.55 ^b^	–	10.20 ± 1.92	**< 0.05**
Intraoperative blood loss (ml)	70.20 ± 36.62 ^a,b^	159.80 ± 28.32 &	137.40 ± 38.16	**< 0.05**
Drain volume (ml)	218.26 ± 102.37 ^a,b^	77.80 ± 26.05	101.40 ± 35.91	**< 0.05**
Total blood loss (ml)	611.02 ± 299.22	695.10 ± 333.43	666.88 ± 485.03	0.53
No. of patients given transfusion (%)	0	0	0	**–**
Postoperative laboratory values				
Postop. Hemoglobin (g/L)	109.32 ± 14.82	104.06 ± 15.17	104.84 ± 13.78	0.15
Postop. Hematocrit (%)	0.33 ± 0.04	0.32 ± 0.05	0.32 ± 0.04	0.32
Post. CRP (mg/L)	126.65 ± 67.41 ^a^	79.08 ± 53.05 &	105.21 ± 26.36	**< 0.05**
Post. ESR (mm/L)	70.64 ± 11.96 ^a^	45.02 ± 10.98 &	66.18 ± 28.27	**< 0.05**
Post. IL-6 (pg/ml)	133.67 ± 46.25 ^a^	77.29 ± 80.65 &	106.75 ± 108.09	**< 0.05**
Post. CK (IU/L)	226.66 ± 103.03 ^a^	158.08 ± 100.72	196.92 ± 93.94	**< 0.05**
Post. Length of hospital stay	4.42 ± 1.23 ^a,b^	3.50 ± 0.54 &	3.70 ± 0.91	**< 0.05**
**Post. Range of motion (3 days)**	**100 ± 5** ^a,b^	**104 ± 6**	**103 ± 6**	**< 0.05**
**Post. Range of motion (3 months)**	**111 ± 9**	**111 ± 8**	**112 ± 9**	**0.90**
Post. VAS (1 day)	3.70 ± 1.18 ^a^	3.08 ± 1.05	3.44 ± 1.11	**0.02**
Post. VAS (3 days)	2.28 ± 0.61 ^a^	1.80 ± 0.53	1.86 ± 0.67	**< 0.05**
Post. HSS score	83.96 ± 3.24	84.16 ± 2.92	83.94 ± 3.71	0.94

*The *p* value represents the result of one-way analysis of variance for independent means for continuous variables that included the three groups. *P* values with statistical significance are in bold print. ^a^ Significantly different from the non-tourniquet group

^b^ Significantly different from the tourniquet in cementation group. & Significantly different from the tourniquet in cementation group

No DVT, PE, or fracture occurred among the groups at the end of follow up. Two cases in the group A and 1 case in the group C were diagnosed with calf muscular venous thrombosis by color Doppler ultrasonography during hospitalization **with no specific treatment**. No lower extremities swelling was found and treated with routine prophylactic anticoagulation treatment. One case in the group A suffered from wound superficial cellulitis, cured with antibiotics and dressing changes.

### Blood loss and transfusion

The mean intraoperative blood loss in the group A, group B, and group C were 70.20 ml, 159.80 ml, and 137.40 ml, respectively, with significant intergroup differences. The mean drain volume was 218.26 ml in the group A, 77.80 ml in the group B, and 101.40 ml in the group C. Group A resulted in significantly higher drain volume compared with the other two groups. The mean hemoglobin and hematocrit was 109.32 g/L, 0.33% in the group A, 104.06 g/L, 0.32% in group B, and 104.84 g/L, 0.32% in the group C, with no significant intergroup differences. Calculated by Gross formulation, the mean total blood loss in the group A, group B, and group C were 611.02 ml, 695.10 ml, and 666.88 ml, respectively, with no significant intergroup differences. No patient received a transfusion in any group.

### Inflammatory factor assessment

The mean post. CRP, ESR, and IL-6 was 126.65 mg/L, 70.64 mm/L, and 133.67 pg/ml in group A, 79.08 mm/L, 45.02 mm/L, and 77.29 pg/ml in group B, 105.21 mg/L, 66.18 mm/L, and 106.75 pg/ml in group C. Group B resulted in significantly less CRP, ESR, and IL-6 levels compared with the other two groups. There was no difference between the group A and **group C** in terms of these three factors. The mean post. CK was 226.66 IU/L in group A, 158.08 IU/L in group B, and 106.92 IU/L in group C, respectively. Group A showed significantly higher CK levels compared to the other two groups.

### Post. Recovery and rehabilitation

The mean length of hospital stay in the group A, group B, and group C was 4.42, 3.50, and 3.70, respectively, with significant intergroup differences.

The mean range of motion at POD 3 days and 3 months were 100° and 111° in the group A, 104°and 111°in the group B, and 103°and 112°in the group C, respectively. Group A resulted in a significantly lower range of motion at POD 3 days compared with the other two groups. However, no significant difference was found in POD 3 months in terms of a range of motion at POD 3 months.

VAS pain scores at 1 and 3 days after operation were 3.70 and 2.28 in the group A, 3.08 and 1.86 in the group B, and 3.44 and 1.80 in the group C, respectively. Group A showed significantly higher pain points at both the two postoperative time points compared with the other two groups.

The mean HSS score was 83.96 in the group A, 84.16 in the group B, and 83.94 in the group C, respectively, with no significant difference among the groups.

### Bone cement mantle thickness assessment

The bone cement mantle thickness in the three groups in different zones was ranged from 0.26 cm to 0.33 cm (Table 3). No significant difference was found among the three groups in each zone. The average bone cement mantle thickness was 0.28 cm in the group A, 0.29 cm in the group B, and 0.29 cm in the group C, which also showed no significant difference.

**Table 3:** Bone cement mantle thickness measurement (cm)

Variable	Group A (Tourniquet group) (*N* = 50)	Group B (Non-tourniquet group) (*N* = 50)	Group C (Tourniquet in cementation group) (***N*** = 50)	*p* value*
Zone 1	0.26 ± 0.07	0.28 ± 0.03	0.27 ± 0.04	0.17
Zone 2	0.26 ± 0.04	0.26 ± 0.04	0.26 ± 0.03	0.24
Zone 3	0.33 ± 0.07	0.33 ± 0.06	0.32 ± 0.08	0.84
Zone 4	0.29 ± 0.06	0.30 ± 0.06	0.29 ± 0.05	0.54
Average	0.28 ± 0.06	0.29 ± 0.07	0.29 ± 0.06	0.56

*The *p* value represents the result of one-way analysis of variance for independent means for continuous variables that

included the three groups

*P* values with statistical significance are in bold print

## Discussion

The most important finding of our present study was that, with the advantage of TXA combined application, performing a tourniquetless TKA may result in less soft tissue injury and rapid postoperative recovery, without increasing blood loss and complication risk.

Table 4 summarized the previous reported results of different tourniquet application strategy in TKA since 2010. According to the survey by AAHKS, approximately 95% of surgeons used a tourniquet in TKA routinely [15]. Advocates of tourniquet believed that the use of a tourniquet is the most effective method for bleeding control. However, opponents of tourniquet pointed out that the application of a tourniquet shows no obvious benefit on the total blood loss because hidden blood loss is a significant portion of total blood loss and should not be ignored [16]. In our study, we found intraoperative blood loss in patients of the tourniquet group was significantly less than patients in non-tourniquet and tourniquet in cementation groups. However, the volume measured in the surgical drain was significantly higher in the tourniquet group. After calculated by Gross formula, no obvious difference was found among the three groups with regard to the total blood loss.

**Table 4:** Previous reported results of different tourniquet application strategy

Author	Location	Patients No.			Tourniquet application strategy	Results	Authors opinion	Study type
		Tourniquet group	Non-tourniquet group	Limited tourniquet group				
Ozkunt 2018	Turkey	24	25	20	Tourniquet usage during the entire operation/no use tourniquet/use of tourniquet at the time of cementation	Long-duration tourniquet use can lead higher pain scores and reduce functional recovery after TKA	Prefer to non-tourniquet	RCT
Zhi 2017	China	94	36	–	Tourniquet utilization lasted from the beginning of cutting bone to the end of binding up	Tourniquet is not associated with reduced blood loss and increased complications	Prefer to tourniquet	Retrospective
Vertullo 2017	Australia	20	20	–	Tourniquet was inflated to 300 mmHg for the duration of the cementing procedure	Tourniquet inflation during cementation does not appear to improve tibial cementation penetration	Prefer to non-tourniquet	RCT
Pfitzner 2016	Germany	45	45	–	Tourniquet was inflated from skin incision until skin closure	Tourniquet application increased cement mantle thickness also increase blood loss and postoperative pain	Neutral opinion	RCT
Dennis 2016	USA	28	28 (not used or only during cementation)		Tourniquet was inflated before the incision and released after cementation	Tourniquet inflation diminished quadriceps strength during the first 3 months	Prefer to non-tourniquet	RCT
Fan 2014	China	30	–	30	Tourniquet was used throughout the surgical procedure or starting during the cementation	Tourniquet limited usage provided the benefits	Prefer to tourniquet	RCT
Tarwala 2014	USA	39	–	36	Use of a tourniquet throughout TKA procedure or only during cementation	Tourniquet inflation for cementation only provide the benefit of bloodless bone for fixation	Prefer to non-tourniquet	RCT
Huang 2014	China	30	–	30 30	Three different tourniquet application strategies	Using a tourniquet full time causes intraoperative blood loss and more excessive inflammation and muscle damage	Prefer to non-tourniquet	RCT
Tai 2012	China	36	36	–	Tourniquet was inflated during operation and released after joint capsule closed	Tourniquet application was effective for reducing blood loss and avoiding inflammation and muscle damage	Prefer to tourniquet	RCT

Abbreviations: RCT, randomized controlled trial

TXA application plays an important role in reducing blood loss for patients receiving TKA without a tourniquet, especially for the combined application strategy. Huang et al. [17] from our institution has proved that TKA patients treated with multiple doses of intravenous and topical TXA had less hidden blood loss, better knee function recovery, and better early satisfaction than controlled patients. Multiple doses of intravenous TXA application can prevent systemic plasminogen activation and delay fibrinolysis, which can result in intraoperative blood loss and postoperative hidden blood loss reduction [18]. Compared with intravenous TXA, topical application can provide a maximum concentration of TXA at the bleeding site, lower TXA absorption, reduce limb swelling, and improve wound-healing [19, 20]. In our study, although the intraoperative blood loss and drain volume were different, there was no significant difference among the three groups in terms of total blood loss. Also, no patient received a transfusion and no VTE complication occurred after TKA. For consideration of TXA advantages, we believe that tourniquet is not necessary when performing a TKA, because the most advantage of tourniquet in reducing blood loss has disappeared [8].

Tourniquet inflation, which is an obvious cause of perioperative hypoxia, can cease blood flow and damage soft tissue by acute ischemia-reperfusion [21]. Platelet, leukocyte, and endothelium activities as well as their interactions were demonstrated to enhance during the perioperative period of TKA, and tourniquet inflation could exaggerate these responses [22]. Lesser tourniquet time is much better for tissue oxygenation and early wound dryness [23]. Our result showed, the soft tissue damage indicators, including CRP, ESR, IL-6, and CK, were significantly lower in patients without tourniquet compared with those in the other two tourniquet groups. Furthermore, VAS scores (1 and 3 days post.), range of motion on 3 days postoperatively and length of hospital stay in tourniquetless group also showed better results than the other two groups. Our results were consistent with other previous clinical studies. Ledin et al. [24] performed a randomized RSA study involving 50 patients and found tourniquet use could cause more postoperative pain and less range of motion. A recent RCT by Dennis et al. [6] demonstrated patients who underwent TKA using a tourniquet had diminished quadriceps strength during the first 3 months after TKA. A meta-analysis by Zhang et al. [1] demonstrated that TKA with a tourniquet might hinder patients’ early postoperative rehabilitation exercise. With the current improvements in surgical techniques and TXA usage strategy, tourniquet application during TKA procedure may decelerate the rapid track recovery rate and increase complication rates, and gradually become unnecessary.

Aseptic loosening is one of the most frequent causes of TKA revision [25, 26]. In order to achieve long term implant stability and survival, creating a clean and blood-poor bone surface for cement penetration is one of the important reasons for tourniquet application. Previous studies have demonstrated that implant stability depends on cement penetration and mantle thickness [27]. Some still worried about the absence of tourniquet might affect cement penetration and implants fixation [7]. However, as the surgical technique develops, some recent studies demonstrated that tourniquet inflation during a TKA procedure does not appear to improve implant cementation penetration or fixation [23, 28]. Our present study showed that there was no significant difference among the three groups in four zones of tibial cement mantle thickness. In our opinion, TXA application and controlled hypotension are effective methods to reduce blood loss and provide a blood-free bone-cement interface, instead of using a tourniquet. Furthermore, to our knowledge, DTS was first applied to determine cement mantle thickness in TKA patients. This new radiographic technique provides good contrast, enhance the visibility of a cement mantle, and remove ghost artifacts in prosthesis patients, which will be more widely used in the joint arthroplasty field [11].

This study has some limitations. First, the 3 months duration of follow-up in the present study might have concealed different long-term outcomes for the tourniquet usage. However, tourniquet mainly influences the early rehabilitation of a TKA patient, without a long-term effect. Second, in order to predict implant survival, we only determined the cement mantle thickness. However, although mantle thickness can influence implant fixation, the relationship between mantle thickness and implant survival is still in controversy. Finally, the study population was small. For the low incidence of postoperative complications, such as infection, DVT, and PE, a larger sample size might be better to find the statistical difference among the groups.

## Conclusions

In conclusion, compared with the other two tourniquet groups, the absence of tourniquet did not appear to affect blood loss and cement penetration in TKA patients. Furthermore, less inflammation reaction and better knee function can be achieved without a tourniquet. For consideration of the effectiveness and safety of tourniquet application, we recommend no longer use a tourniquet in primary TKA when TXA is administrated. More high-quality studies with larger number of participants and longer follow-up are needed to confirm the effect of tourniquet on clinical outcomes of knee OA patients treated with TKA.

## Data Availability

Request for details in the study dataset can be submitted to the corresponding author. Human subject protection requirements, appropriate data privacy as well as institutional requirements must be met.
